# Derivation and validation of novel integrated inpatient mortality prediction score for COVID-19 (IMPACT) using clinical, laboratory, and AI—processed radiological parameter upon admission: a multicentre study

**DOI:** 10.1038/s41598-023-50564-9

**Published:** 2024-01-25

**Authors:** Eric Daniel Tenda, Joshua Henrina, Andry Setiadharma, Dahliana Jessica Aristy, Pradana Zaky Romadhon, Harik Firman Thahadian, Bagus Aulia Mahdi, Imam Manggalya Adhikara, Erika Marfiani, Satriyo Dwi Suryantoro, Reyhan Eddy Yunus, Prasandhya Astagiri Yusuf

**Affiliations:** 1 Pulmonology and Critical Care Medicine Division, Department of Internal Medicine, Dr. Cipto Mangunkusumo National Referral Hospital, Faculty of Medicine Universitas Indonesia, Jl. Pangeran Diponegoro No. 71, RW. 5, Kenari, Kec. Senen, Kota Jakarta Pusat, Daerah Khusus Ibukota Jakarta, 10430 Indonesia; 2 Medical Technology Cluster of Indonesian Medical Research Institute (IMERI), Faculty of Medicine Universitas Indonesia, Jakarta, Indonesia; 3 Hematology and Medical Oncology, Department of Internal Medicine, Universitas Airlangga Academic Hospital, Faculty of Medicine Universitas Airlangga, Surabaya, Indonesia; 4 Pulmonology and Critical Care Medicine Division, Department of Internal Medicine, Faculty of Medicine, Public Health and Nursing, Universitas Gadjah Mada, Dr. Sardjito General Hospital, Yogyakarta, Indonesia; 5 Department of Internal Medicine, Faculty of Medicine Universitas Airlangga, Surabaya, Indonesia; 6 Cardiology Division, Department of Internal Medicine, Faculty of Medicine, Public Health and Nursing, Universitas Gadjah Mada, Dr. Sardjito General Hospital, Yogyakarta, Indonesia; 7 Pulmonology and Critical Care Medicine Division, Department of Internal Medicine, Faculty of Medicine Universitas Airlangga, Universitas Airlangga Academic Hospital, Surabaya, Indonesia; 8 Nephrology Division, Department of Internal Medicine, Faculty of Medicine Universitas Airlangga, Universitas Airlangga Academic Hospital, Surabaya, Indonesia; 9 Department of Radiology, Dr. Cipto Mangunkusumo National Referral Hospital, Faculty of Medicine Universitas Indonesia, Jakarta, Indonesia; 10 Department of Medical Physiology and Biophysics, Faculty of Medicine Universitas Indonesia, Jakarta, Indonesia

**Keywords:** Infectious diseases, Viral infection, Medical imaging, Prognosis

## Abstract

Limited studies explore the use of AI for COVID-19 prognostication. This study investigates the relationship between AI-aided radiographic parameters, clinical and laboratory data, and mortality in hospitalized COVID-19 patients. We conducted a multicentre retrospective study. The derivation and validation cohort comprised of 512 and 137 confirmed COVID-19 patients, respectively. Variable selection for constructing an in-hospital mortality scoring model was performed using the least absolute shrinkage and selection operator, followed by logistic regression. The accuracy of the scoring model was assessed using the area under the receiver operating characteristic curve. The final model included eight variables: anosmia (OR: 0.280; 95%CI 0.095–0.826), dyspnoea (OR: 1.684; 95%CI 1.049–2.705), loss of consciousness (OR: 4.593; 95%CI 1.702–12.396), mean arterial pressure (OR: 0.928; 95%CI 0.900–0.957), peripheral oxygen saturation (OR: 0.981; 95%CI 0.967–0.996), neutrophil % (OR: 1.034; 95%CI 1.013–1.055), serum urea (OR: 1.018; 95%CI 1.010–1.026), affected lung area score (OR: 1.026; 95%CI 1.014–1.038). The Integrated Inpatient Mortality Prediction Score for COVID-19 (IMPACT) demonstrated a predictive value of 0.815 (95% CI 0.774–0.856) in the derivation cohort. Internal validation resulted in an AUROC of 0.770 (95% CI 0.661–0.879). Our study provides valuable evidence of the real-world application of AI in clinical settings. However, it is imperative to conduct prospective validation of our findings, preferably utilizing a control group and extending the application to broader populations.

## Introduction

More than 3 years since the first Coronavirus disease 2019 (COVID-19) appeared in Wuhan, China, and started a once-in-a-century pandemic^1^. While advancement in the treatment and prevention of severe COVID-19 disease has progressed rapidly, this respiratory viral disease remains an important source of worldwide morbidity and mortality^2^. A new wave of cases is still being reported due to viral mutation, partly due to antivirals and less efficacious vaccines, which selectively produce more resistant strains. Learning from the constraints caused by the COVID-19 waves, a clinical decision tool is essential for managing outbreaks of COVID-19 cases to help in triaging patients and thus preventing scarcity of hospital beds and medical resources^3^.

Numerous clinical decision tools have been created and published for the purpose mentioned above, such as the COVID-GRAM and the 4C Mortality score^4,5^. To the best of our knowledge, the clinical decision tools available today utilize clinical parameters and laboratory data only. It is understandable because these tools must be simple and practical and have adequate accuracy to be used clinically. Understandably, there are no clinical decision tools that incorporate radiographic AI parameter of COVID-19 patients.

The role of artificial intelligence (AI) in the medical field has expanded rapidly. Mainly, this role is limited to the purpose of screening and diagnosis. For example, in the field of pulmonology, AI-aided radiographic interpretation of chest X-ray (CXR) images proved to be sensitive and accurate for pulmonary tuberculosis screening^6^. The role of AI in COVID-19 diagnosis has also been reported. In one study, CAD4COVID-Xray (an AI software), through the color heatmap method, had a superior COVID-19 pneumonia diagnosis compared to six radiologists^7,8^. In contrast, the evidence on incorporating AI-aided radiographic interpretation of CXR for predicting clinical outcomes is scarce.

This study aimed to investigate the relationship between AI-aided radiographic parameter, clinical and laboratory data, and clinical outcomes in hospitalized COVID-19 patients with confirmed RT-PCR results. Additionally, we aimed to develop and validate a clinical risk tool known as Integrated Inpatient Mortality Prediction Score for COVID-19 (IMPACT) by integrating these data.

## Methods

### Study design

This was a retrospective cohort study using a secondary data from medical records and Picture Archiving and Communication System (PACS) chest radiography repositories. This study was conducted at three academic hospital, i.e., Airlangga University Hospital, located in Surabaya, East Java Province, Sardjito General Hospital, located in Jogjakarta, Special Region of Jogjakarta Province, and in dr. Cipto Mangunkusumo General Hospital, located in Centre Jakarta, Special Capital Region of Jakarta. The ethics committee of the respective hospitals approved the study. The requirement for written informed consent was exempted due to the utilization of anonymized historical data. (University of Gadjah Mada, University of Airlangga, and University of Indonesia IRB). All methods were performed in accordance with the relevant guidelines and regulations and adhered to Declaration of Helsinki.

Data obtained from the first two hospitals was used for the derivation of the novel scoring system. Data obtained from the latter hospital was used for the validation of the novel scoring system.

This study enrolled a cohort of adult patients (≥ 18 years old) hospitalized with COVID-19 cases between April 2020 and April 2022 for the derivation cohort. The validation cohort, however, included only hospitalized COVID-19 cases from April 2020 to April 2021. The reasons for this approach are twofold. Firstly, we utilized data from a separate study to constitute the validation cohort. Secondly, due to the expiration of our software permit, we were unable to access the CXR software required for additional analysis. As a result, we relied solely on the available data for analysis.

The decision to utilize chest X-ray rather than more advanced imaging modalities is driven by two primary considerations. First and foremost is Indonesia's classification as a low to middle-income country (LMIC). The accessibility of chest CT-scans is constrained, predominantly concentrated in major cities, reflecting the uneven distribution of healthcare infrastructure. Furthermore, adhering to WHO guidelines, chest radiography provides higher specificity and can be performed using portable equipment at the point of care. This not only tackles the issue of restricted access but also mitigates the risk of cross-infection linked to patient transport^9,10^.

COVID-19 cases were verified using either positive high-throughput sequencing or real-time reverse-transcription polymerase-chain-reaction (RT-PCR) tests conducted on nasal and pharyngeal swab samples.

This study excluded cases with substandard chest radiography qualities, large lung cavities on CXR, concurrent mediastinal or lung mass, and an interval between RT-PCR and CXR acquisition of more than 7 days.

Included in the data collection including the patients’ demographic data, vaccination data, comorbidities, clinical and laboratory data in the emergency department, treatment, and discharge outcome. COVID-19 disease severity was determined on hospital admission and was stratified according to the local Indonesian Guideline, which adopted the WHO COVID-19 disease severity stratification^11^.

A group of internal medicine physicians carefully examined, summarized, and verified the data. Two clinicians independently reviewed each record.

### Potential predictive variables

We included the following variables: gender, vaccination status, smoking history, and comorbidities (autoimmunity, obesity, arterial hypertension, diabetes mellitus, asthma, coronary heart disease, cerebrovascular disease, chronic obstructive pulmonary disease, pulmonary tuberculosis, chronic kidney disease, chronic liver disease, brain disease, immunodeficiency disease, and cancer), as well as symptoms (fever, cough, sore throat, rhinorrhea, anosmia, myalgia, headache, malaise, anorexia, diarrhea, nausea, vomiting, abdominal pain, dyspnoea, chest pain, loss of consciousness), CXR data (CXR projection, pneumonia on CXR, CXR AI probability score, CXR AI affected lung area [ALA] score), sepsis, septic shock, ARDS, co-infection, clinical data (systolic blood pressure, diastolic blood pressure, pulse rate, temperature, respiratory rate, peripheral oxygen saturation, and symptom onset), and laboratory data (haemoglobin, potassium, sodium, white blood cells, lymphocytes, neutrophil levels, thrombocyte count, neutrophil-to-lymphocyte ratio, and urea levels).

### Outcomes

The primary outcome of this study was in-hospital mortality. The secondary outcome of this study was disease progression defined as at least one degree increment of disease severity (e.g., disease progression from moderate to severe disease).

### AI system for chest X-ray interpretation

CAD4COVID-Xray software (Thirona, Nijmegen, Netherlands; https://covid.cad4tb.care/accounts/login/?next=/; based on CAD4TB ver. 6) was used for AI chest X-ray interpretation. The principal objective of the CAD4COVID-Xray software is to facilitate the triaging process in environments with limited resources and in areas with a high prevalence of COVID-19. This product holds a CE certification and employs the identical technical core utilized by CAD4TB, another CE-certified product registered by the FDA in Ghana. Consequently, CAD4COVID-Xray is developed to the same high-quality standard as CAD4TB, a standard substantiated by validation through over 40 academic publications. CAD4TB has been successfully deployed in 35 countries, playing a pivotal role in screening six million people globally^12^. Hence, its reliability, pertinence, and applicability have undergone rigorous validation, affirming that CAD4COVID-Xray is not only a dependable solution but also possesses relevance and generalizability across diverse healthcare scenarios.

**Figure 1:**
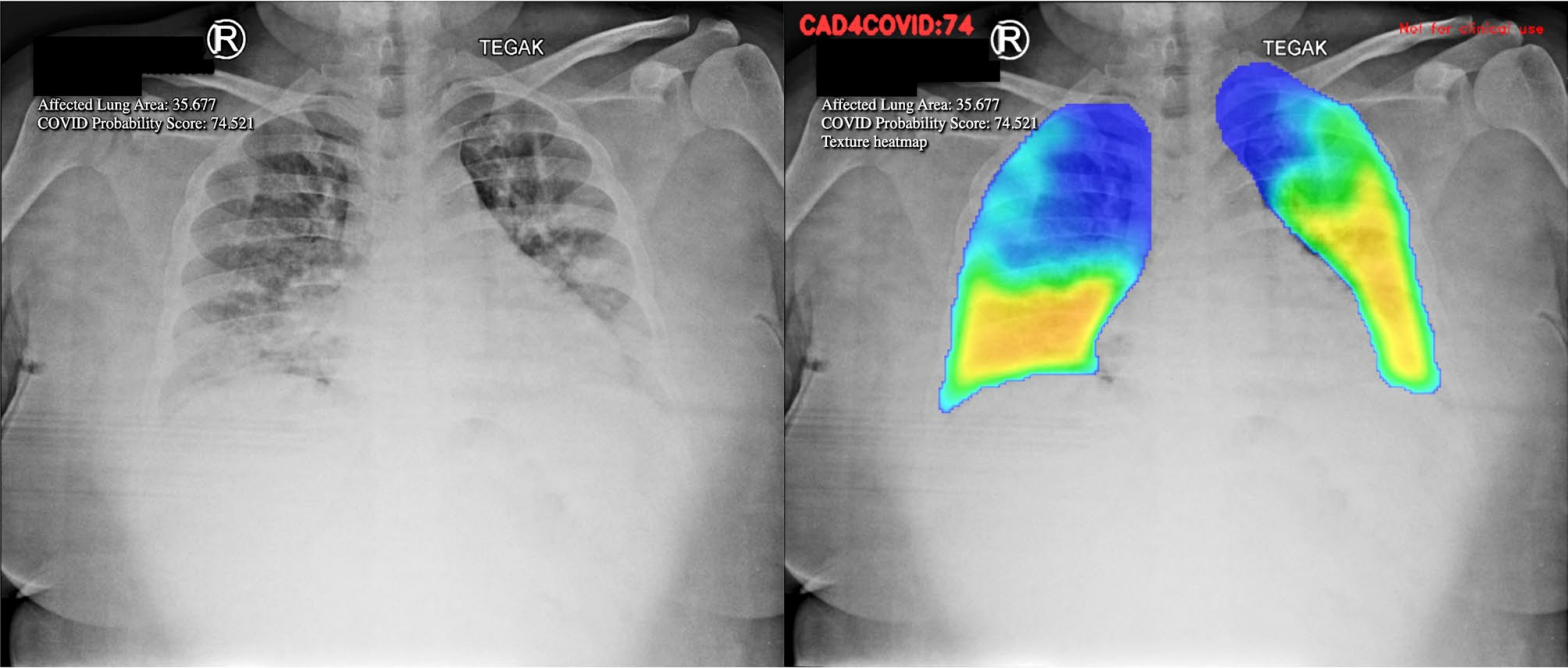
Detection of lung parenchymal abnormality in the chest X-ray with the heatmap method.

The AI software relies on color heat-map method to detect parenchymal abnormality on chest X-rays (Fig. 1).

The software executed a series of steps outlined below:

1. Pseudoanonymization of COVID-19 Patients’ Digital Chest X-ray
  Confirmed COVID-19 patients' digital chest X-rays (in DICOM format) underwent pseudoanonymization before being uploaded using the PACS INFINITT software from INFINITT Healthcare in Seoul, South Korea.
2. Chest X-ray scoring process
  The chest X-ray scoring involved four consecutive steps:
  Normalization: Standardizing the CXR scale for consistent handling by the AI and generalization from CXRs of different sizes.
  Segmentation of Lung Fields: Automatic separation of lungs from the surrounding areas of the image by the AI.
  Texture Evaluation: Identification of relevant anomalies in the lung segments.
  Area Analysis: Determination of the proportion of lung parenchyma affected.
3. Filter Weight Determination
  The weights of each filter were determined, and an average filter weight was applied as a mask on the CXR picture. This created a color heat map visible only on the lung area previously segregated by the trained model.
4. Color Heat Map Representation:
  The color heat map exhibited various colors based on data weight:
  High, medium, low, and extremely low probabilities of abnormality were represented by the colors red, yellow, green, and blue, respectively.
5. CAD4COVID Software Scoring:
  The CAD4COVID software received the digital CXR file and generated two AI scorings:
  Affected Lung Area (ALA) Score: Calculated from the total lung volume with abnormalities found on the CXR, ranging from 0 to 100. A higher score indicates a greater impact on lung tissue.
  COVID Probability Score: Determined by the average final weight of all layers, ranging from 0 to 100. A higher score suggests a higher likelihood of COVID-19 occurrence.

### Variable selection and establishing a scoring system

For the variable selection and scoring system derivation, we included all 512 hospitalized COVID-19 patients in the derivation cohort. In the selection process, we entered 66 variables. We applied the Least Absolute Shrinkage and Selection Operator (LASSO) regression with the purpose to minimize the potential collinearity of measured variables from the same patient and to prevent variables over-fitting. To deal with missing values, imputation was considered if the missing values were less than 25%. We used Multiple Imputation by Chained Equations (MICE) to impute numeric, binary, and factor variables^13^. In our multivariable analyses, we used least absolute shrinkage and selection regression with L1 penalization and tenfold cross-validation for internal validation^4^. Based on its value, this logistic regression model imposes penalties on the absolute magnitudes of the regression coefficients. The estimates of weaker components are minimized towards zero by using greater penalties, leaving just the most significant predictors in the model. The factors that were shown to be the most predictive were those with the lowest value (min). The LASSO regression was carried out using the statistical program “glmnet” from the R Foundation. The risk score was then created using the consistently statistically significant factors that were included in logistic regression models after the variables found by LASSO regression analysis were included. (supplementary file).

### Accuracy assessment

The IMPACT’s score accuracy was assessed using the area under the receiver-operator characteristic curve (AUROC). Statistical analysis was performed with the IBM Statistical Program for Social Science (SPSS) for Macintosh, 27.0 (IBM Corp., Armonk, NY, USA) with statistical significant set at *P* < 0.05.

### IMPACT score validation

The IMPACT score was validated using data from Dr. Cipto Mangunkusumo General Hospital (RSCM), which included a cohort of 137 patients. RSCM is a national referral hospital situated in Jakarta, the capital city of Indonesia. As a result, the baseline characteristics of hospitalized COVID-19 patients in this study were highly diverse, showcasing the ethnic and racial heterogeneity of Indonesia. The data collected from RSCM underwent meticulous scrutiny and verification by two physicians (AS and JH). This dataset was utilized to compute the IMPACT COVID-19 mortality risk score, as mentioned earlier, for the derivation cohort.

### Characteristics of derivation cohort

In the derivation cohort, we included a total of 512 patients from two academic medical centres located in Surabaya, East Java Province, and the Special Region of Jogjakarta, Jogjakarta Province, spanning from April 2020 to April 2022. Upon hospital admission, the proportion of COVID-19 disease severity, as per the Indonesian COVID-19 guideline, was as follows: mild cases accounted for 5.9%, moderate cases for 41.2%, severe cases for 30.9%, and critical cases for 22.1%. Out of 512 patients, 106 patients (20.7%) experienced clinical worsening during their hospitalization, resulting in an in-hospital mortality rate of 28.9%. The top five pre-existing conditions observed in these patients were hypertension, diabetes, obesity, coronary heart disease, and chronic kidney disease. The predominant symptoms reported were cough, fever, dyspnoea, nausea, and malaise (refer to Table 2). Abnormal chest X-rays were identified in 412 (80.47%) patients, and further details regarding laboratory findings can be found in Table 2.

### Predictor selection

**Figure 2:**
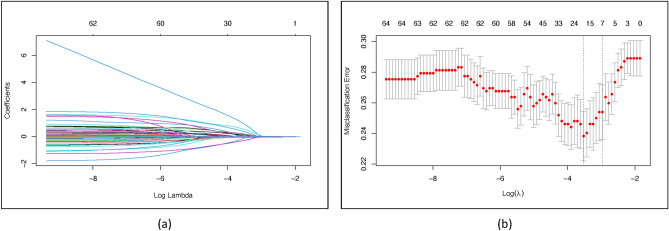
Variable selection for model construction using the least absolute shrinkage and selection operator (LASSO) binary logistic regression model. (**a**) LASSO coefficient profiles of the 66 baseline variables. (**b**) Tuning parameter selection for the LASSO model using tenfold cross-validation and minimum criteria.

For the LASSO regression, we entered 66 variables measured at hospital admission (Tables 1 and 2). Through LASSO regression with, we identified 18 variables that were significant predictors for mortality (Fig. 2). These variables were gender, vaccination status, positive cancer status, sore throat, anosmia, dyspnoea, lost of consciousness, effusion on CXR, septic shock, CXR ALA score, mean arterial pressure, respiratory rate, peripheral oxygen saturation, white blood cells count, neutrophil count, neutrophil–lymphocyte-ratio, serum urea levels, and potassium levels. Subsequently, these variables were entered in logistic regression. The final variables included in final model were anosmia (OR: 0.280; 95%CI 0.095–0.826; *P* = 0.021), dyspnoea (OR: 1.684; 95%CI 1.049–2.705; *P* < 0.031), loss of consciousness (OR: 4.593; 95%CI 1.702–12.396; *P* = 0.003), mean arterial pressure (OR: 0.928; 95%CI 0.900–0.957; *P* = 0.007), peripheral oxygen saturation (OR: 0.981; 95%CI 0.967–0.996; *P* = 0.012), neutrophil % (OR: 1.034; 95%CI 1.013–1.055; *P* = 0.001), serum urea (OR: 1.018; 95%CI 1.010–1.026; *P* < 0.001), affected lung area score (OR: 1.026; 95%CI 1.014–1.038; *P* < 0.001). (Table 3).

**Table 1:** Demographic and clinical characteristics of the derivation cohort.

Baseline characteristics	Derivation cohort (n = 512)	Mortality	
		Survived (n = 364)	Deceased (n = 148)
Age, years	55 (44–64)	54 (41–63)	58 (50–65)
Male	260 (50.8)	169 (46.4)	91 (61.5)
BMI, kg/m^2^	24.22 (22.25–27.18)	24.44 (22.23–27.06)	23.88 (22.32–27.68)
Current smokers	25 (6.8)	23 (6.3)	12 (8.1)
Symptoms onset, days	4 (2–7)	4 (2–7)	5 (3–7)
Intubation on admission	7 (1.4)	3 (0.8)	4 (2.7)
Length of stay, days	9.00 (5.25–13.00)	10.00 (7.00–14.00)	6.00 (3.00–10.75)
Disease severity			
Mild	30 (5.9)	29 (8.0)	1 (0.7)
Moderate	211 (41.2)	168 (46.2)	43 (29.1)
Severe	158 (30.9)	96 (26.4)	62 (41.9)
Critically ill	113 (22.1)	71 (19.5)	42 (28.4)
Complications and coincidences			
Sepsis	65 (12.7)	44 (12.1)	21 (14.2)
Septic shock	46 (9.0)	24 (6.6)	22 (14.9)
ARDS	79 (15.4)	47 (12.9)	32 (21.6)
Secondary infection	70 (13.7)	48 (13.2)	22 (14.9)
Comorbidities			
No. of comorbidities			
0	130 (25.4)	100 (27.5)	30 (20.3)
1	174 (34.0)	124 (34.1)	50 (33.8)
2	120 (23.4)	84 (23.1)	36 (24.3)
3	50 (9.8)	34 (9.3)	16 (10.8)
4	24 (4.7)	14 (3.8)	10 (6.8)
5	10 (2.0	7 (1.9)	3 (2.0)
6	3 (0.6)	1 (0.3)	2 (1.4)
7	1 (0.2)	0 (0.0)	1 (0.7)
Hypertension	187 (36.5)	131 (36.0)	56 (37.8)
Diabetes mellitus	187 (36.5)	125 (34.3)	62 (41.9)
Obesity	122 (23.8)	91 (25.0)	31 (20.9)
Coronary heart disease	67 (13.1)	43 (11.8)	24 (16.2)
Chronic kidney disease	47 (9.2)	25 (6.9)	22 (14.9)
Congestive heart failure	21 (4.1)	11 (3.0)	10 (6.8)
Cancer	20 (3.9)	10 2.7)	10 (6.8)
Cerebrovascular disease	14 (2.7)	10 (2.7)	4 (2.7)
Asthma	10 (2.0)	7 (1.9)	3 (2.0)
COPD	8 (1.6)	5 (1.4)	3 (2.0)
Pulmonary tuberculosis	6 (1.2)	3 (0.8)	3 (2.0)
Autoimmune	5 (1.0)	4 (1.1)	1 (0.7)
Chronic liver disease	3 (0.6)	1 (0.3)	2 (1.4)
HIV/AIDS	3 (0.6)	2 (0.5)	1 (0.7)

Categorical data are summarized using frequencies and percentages n (%). Continuous data are presented either as mean values with standard deviation (mean ± SD) for normally distributed data, or as median values with the first and third quartiles [median (Q1–Q3)] for data that are not normally distributed. *BMI* Body Mass Index, *ARDS* acute respiratory distress syndrome, *COPD* chronic obstructive pulmonary disease, *HIV* human immunodeficiency virus, *AIDS* acquired immunodeficiency syndrome.

**Table 2:** Clinical, laboratory and radiological findings of the derivation cohort (n[%]; mean ± SD; median [Q1–Q3]).

Baseline characteristics	Derivation cohort (n = 512)	Mortality	
		Survived (n = 364)	Deceased (n = 148)
Signs and symptoms			
Cough	346 (67.6)	250 (68.7)	96 (64.9)
Fever	283 (55.3)	204 (56.0)	79 (53.4)
Dyspnoea	261 (51.0)	168 (46.2)	93 (62.8)
Nausea	114 (22.3)	77 (21.2)	37 (25.0)
Malaise	101 (19.7)	70 (19.2)	31 (20.9)
Vomiting	89 (17.4)	57 (15.7)	32 (21.6)
Anorexia	80 (15.6)	52 (14.3)	28 (18.9)
Headache	60 (11.7)	47 (12.9)	13 (8.8)
Rhinorrhea	57 (11.1)	44 (12.1)	13 (8.8)
Anosmia/hyposmia	53 (10.4)	48 (13.2)	5 (3.4)
Diarrhea	45 (8.8)	30 (8.2)	15 (10.1)
Myalgia	37 (7.2)	28 (7.7)	9 (6.1)
Abdominal pain	32 (6.3)	18 (4.9)	14 (9.5)
Sore throat	25 (4.9)	23 (6.3)	2 (1.4)
Loss of consciousness	22 (4.3)	9 (2.5)	13 (8.8)
Chest pain	17 (3.3)	12 (3.3)	5 (3.4)
Vital signs			
Systolic blood pressure, mmHg	128.50 (113.25–142.00)	12.50 (117.00–142.00)	128.50 (110.00–145.00)
Diastolic blood pressure, mmHg	80.00 (70.00–87.00)	80.00 (72.00–87.75)	79.00 (64.75–87.00)
Mean arterial pressure, mmHg	95.00 (86.75–105.58)	95.17 (87.67–105.33)	94.50 (81.85–105.92)
Pulse rate, beats/min	97 (85–108)	96 (84–105)	100 (88–113)
Respiratory rate, breaths/min	24 (20–26)	23 (20–25)	24 (22–28)
Temperature, °C	36.50 (36.00–37.00)	36.50 (36.00–37.00)	36.55 (36.00–37.00)
Peripheral oxygen saturation, %	95.00 (89.00–98.00)	96.00 (91.00–98.00)	89.50 (82.25–96.00)
Laboratory findings			
Haemoglobin, g/dL	13.10 (11.60–14.50)	13.10 (11.70–14.48)	12.80 (11.50–14.68)
Leukocyte, 10^3^ cell/µL	8.32 (5.87–11.44)	8.05 (5.76–10.60)	9.81 (6.45–14.41)
Neutrophil, %	77.75 (69.30–85.78)	75.65 (66.65–83.80)	83.80 (76.93–89.78)
Lymphocyte, %	14.25 (8.50–20.78)	16.15 (9.40–22.48)	10.70 (5.70–17.28)
Neutrophil lymphocyte ratio	5.36 (3.32–9.91)	4.64 (3.02–8.61)	7.83 (4.38–15.85)
Thrombocyte, 10^3^ cell/µL	243.50 (176.00–322.75)	251.50 (183.25–330.75)	234.50 (168.50–308.00)
Serum urea, mg/dL	15.10 (10.50–26.20)	13.60 (9.70–22.08)	23.75 (13.70–48.88)
Sodium, mmol/L	135.00 (131.00–138.00)	135.00 (132.00–138.00)	134.85 (131.00–138.00)
Potassium, mmol/L	4.02 (3.66–4.47)	4.00 (3.64–4.34)	4.12 (3.68–4.74)
Radiological findings			
Radiological pneumonia			
Unilateral	11 (2.1)	8 (2.2)	3 (2.0)
Bilateral	401 (78.3)	274 (75.3)	127 (85.8)
Pleural effusion	6 (1.2)	1 (0.3)	5 (3.4)
AI processed parameter			
Affected lung area score, %	15.65 (2.92–33.65)	9.87 (1.75–28.13)	28.47 (10.83–42.60)
Probability score, %	81.70 (59.02–98.82)	73.50 (56.24–98.14)	93.88 (63.62–99.48)

Categorical data are summarized using frequencies and percentages n (%). Continuous data are presented either as mean values with standard deviation (mean ± SD) for normally distributed data, or as median values with the first and third quartiles [median (Q1–Q3)] for data that are not normally distributed. *AI* artificial intelligence.

**Table 3:** Multivariate logistic regression model for predicting in-hospital mortality 512 COVID-19 inpatients.

Variables	B	OR (95% CI)	*P* value
Anosmia/hyposmia	− 1.273	0.280 (0.095–0.826)	0.021
Dyspnoea	0.521	1.684 (1.049–2.705)	0.031
Loss of consciousness, GCS	1.525	4.593 (1.702–12.396)	0.003
Mean arterial pressure, mmHg	− 0.019	0.928 (0.900–0.957)	0.000
Peripheral oxygen saturation, %	− 0.074	0.981 (0.967–0.996)	0.012
Neutrophil, %	0.033	1.034 (1.013–1.055)	0.001
Serum urea, mg/dL	0.018	1.018 (1.010–1.026)	< 0.001
Affected lung area score, %	0.026	1.026 (1.014–1.038)	< 0.001
Constant	3.749	42.480	0.036

*OR* odds ratio, *GCS* glasgow coma scale.

### IMPACT mortality score construction

The IMPACT score was constructed based on the rounding of the beta coefficients acquired from the logistic model. The mortality risk score was developed by utilizing coefficients from the logistic model. We employed the following formulas in the logistic model to compute the probability and 95% confidence intervals^14^: The probability formula is given by exp(β × X)/[1 + exp(β × X)]. The formula for calculating the lower limit of the 95% confidence interval is exp[Xn × βn—z × SE(β)]/{1 + exp[Xn × βn—z × SE(β)]}, and the formula for calculating the upper limit of the 95% confidence interval is exp[Xn × βn + z × SE(β)]/{1 + exp[Xn × βn + z × SE(β)]}.

Therefore, the formula for calculating the mortality risk is as follows: mortality risk = 100 * (1/(1 + exp(−x))), where (x) is derived from the equation: (Anosmia*−1.273) + (Dyspnoea*0.521) + (Loss of Consciousness*1.525) + (SpO2*-0.074) + (MAP*−0.019) + (Neutrophil Percentage*0.033) + (Serum Urea*0.018) + (ALA Score*0.026) + 3.749.

### IMPACT mortality score performance

**Figure 3:**
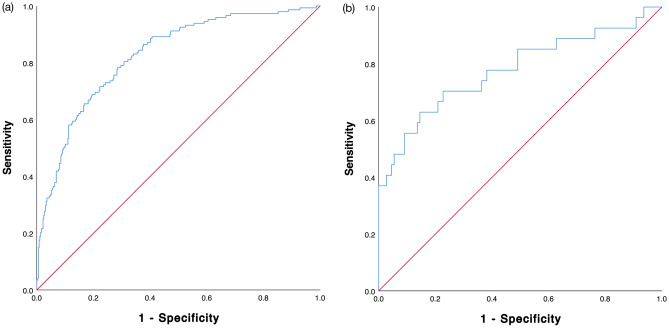
IMPACT in-hospital mortality scoring system area under the receiver—operating characteristic (AUROC) curve: (**a**) Derivation cohort, (**b**) validation cohort.

The predictive value for IMPACT score from the derivation cohort was 0.815 (95% CI 0.774–0.856), which was categorized as a model with a good predictive ability (Fig. 3 panel a). The AUROCs of IMPACT sore for Airlangga University Hospital and dr. Sardjito General Hospital were 0.839 (95%CI 0.768–0.910) and 0.793 (95%CI 0.741–0.845), respectively.

### IMPACT mortality score validation

The internal validation of IMPACT score in the RSCM hospital yielded AUROC score of 0.770 (95%CI 0.661–0.879) with a fair predictive ability (Fig. 3 panel b). The baseline and clinical characteristics of the validation cohort are presented in Table 4.

**Table 4:** Baseline characteristics of the validation cohort (n[%]; mean ± SD; median [Q1–Q3]).

Baseline characteristics	Validation cohort (n = 137)	Mortality	
		Survived (n = 110)	Deceased (n = 27)
Anosmia/hyposmia	25 (18.2)	22 (20.0)	3 (11.1)
Dyspnoea	79 (57.7)	57 (51.8)	22 (81.5)
Loss of consciousness	12 (8.8)	3 (2.7)	9 (33.3)
Mean arterial pressure, mmHg	95.09 ± 15.09	96.40 ± 13.63	89.73 ± 19.37
Peripheral oxygen saturation, %	97.00 (92.00–98.00)	97.00 (93.75–98.00)	95.00 (85.00–98.00)
Neutrophil, %	72.15 ± 13.65	70.39 ± 13.29	79.34 ± 12.94
Serum urea, mg/dL	25.60 (17.70–44.85)	24.50 (17.38–36.75)	47.70 (24.40–120.60)
Affected lung area score, %	8.0 (1.0–26.5)	7.0 (1.0–20)	31.0 (2.0–45.0)

Categorical data are summarized using frequencies and percentages n (%). Continuous data are presented either as mean values with standard deviation (mean ± SD) for normally distributed data, or as median values with the first and third quartiles [median (Q1–Q3)] for data that are not normally distributed.

**Figure 4:**
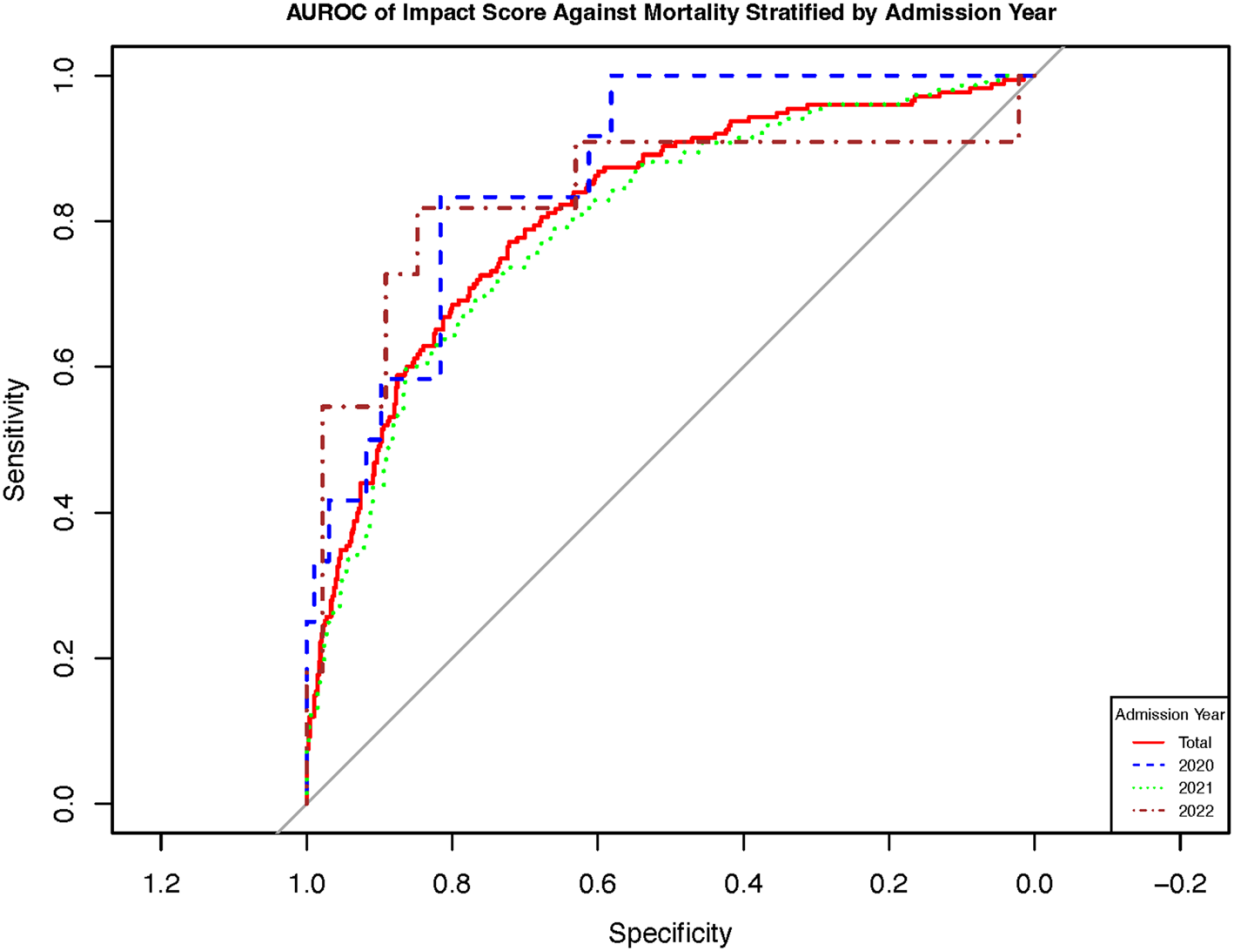
Area under the receiver—operating characteristic of impact score stratified according to the Admission Year.

As previously stated in the Methods section, the disparity in the hospitalization periods between the validation cohort and derivation cohort for COVID-19 patients could raise concerns about the validity of our findings. To address this issue, we conducted a stratified analysis of the AUROC of the IMPACT score based on the year of admission in the derivation cohort. Our analysis revealed no significant differences in the AUROC among the admission years 2020, 2021, and 2022 (Fig. 4 and supplementary material Table 1).

### Ethics approval and consent to participate

Ethical approval for this study was obtained from the University of Gadjah Mada (IRB Approval Number: KE/FK/0900/EC/2022), the University of Airlangga (IRB Approval Number: 073/KEP/2022), and the University of Indonesia’s Faculty of Medicine Internal Review Board (IRB Approval Number: KET-1334/UN2.F1/ETIK/PPM.00.02/2022). The internal review board waived the consent to participate due to the retrospective nature of this study.

## Discussion

In this study, we developed and internally validated the IMPACT Mortality score. The AUROC scores obtained from the derivation and validation cohorts were 0.815 and 0.770, respectively. To the best of our knowledge, this is the first scoring system that integrates clinical and laboratory data with AI-related parameters.

The developed model offers two advantages. Firstly, the scoring system incorporates basic laboratory indices that are accessible even in primary care clinics and small hospitals. This sets it apart from other scoring systems like COVID-Gram, which utilize numerous laboratory parameters that are often unavailable in small/rural hospitals, especially in developing countries. Secondly, the model includes an AI-based parameter called the ALA score, derived from analysed chest X-rays (CXRs). The ALA score is generated by the CAD4COVID software, providing rapid results after uploading the image to the cloud-based system.

While CT-scan is the preferred imaging modality for aiding COVID-19 diagnosis, it may not be accessible in many developing countries. On the other hand, CXR is readily available even in small hospitals. Additionally, logistical challenges such as patient transportation and the need for resuscitation in severe cases make CT-scan impractical^15^.

In the final model, several variables were identified as protective against in-hospital mortality due to COVID-19. These variables include mean arterial pressure (MAP), peripheral oxygen saturation (SpO2), and anosmia. Higher MAP and SpO2 as protective variables may indicate a milder course of COVID-19^16^.

Anosmia is considered a protective factor against in-hospital mortality of COVID-19 disease. A retrospective study involving 576 patients found that those with anosmia had higher levels of lymphocytes, haemoglobin, and GFR, and lower levels of D-dimer and CRP, indicating a milder immune and inflammatory response to SARS-CoV-2 infection^17^. Hendawy et al. also showed that anosmia is associated with mild chest infection^18^.

Conversely, our model identified several risk factors for mortality, including dyspnoea, loss of consciousness (LOC), higher neutrophil count, and higher serum urea levels. These factors are well-known indicators of poor prognosis, even in community-acquired pneumonia^19,20^. Significantly, elevated neutrophil levels contribute to COVID-19-associated coagulopathy by activating neutrophil extracellular traps (NETs)^21^. NETosis, the process where neutrophils release DNA structures to trap and kill pathogens, plays a crucial role in the immune response against infections, including viral infections. Excessive NET release leads to inflammation, tissue damage, and contributes to the cytokine storm observed in severe cases.

Notably, our study emphasizes the importance of quantifying parenchymal abnormalities with the help of an AI-based parameter for the purpose of determining mortality risk.

Many studies have evaluated the diagnostic ability of artificial intelligence against human readers. For example, Murphy et al. found that CAD4COVID X-ray which was trained on 24,678 radiographs, including 1540 radiographs for validation, had a comparable performance against six radiologists^8^.

Another study by Kapoor et al. showed that AI utilization improved triaging system of COVID-19 cases in the emergency department when faced with patients presenting with Flu-like symptoms. Combined with the high resolution CT AI analysis, CXR AI analysis had 97.9% sensitivity and 99% specificity^22^.

In another study, AI parameters derived from CT-scan namely CT—severity score (CT-SS) and affected lung area (%AA)^23^. These parameters associated with poor outcomes such as length of stay, risk of ICU admission, ICU LOS, and risk of mechanical ventilation. The CT-SS had a good predictive ability for ICU admission in COVID-19 patients (AUROC = 0.84; 95%CI 0.79–0.90).

In contrast, the evidence supporting the use of artificial intelligence for disease prognosis is limited. To date, only one published study has specifically investigated this area. In their study, Shamout et al. developed a prognostic model using an artificial intelligence system^24^. The model demonstrated the ability to predict deterioration within 96 h, achieving an AUROC of 0.786 (95% CI 0.745–0.830). To construct the model, the AI system utilized a dataset comprising clinical variables extracted from electronic health records and CXR images. Notably, the model effectively estimated the temporal risk evolution and considered relevant clinical endpoints, such as ICU admission, intubation, and in-hospital mortality.

Comparatively, our model and the model by Shamout et al. differ in terms of the clinical variables incorporated. Their model encompassed a larger set of clinical variables, potentially presenting challenges for replication in developing countries. In contrast, our model included a greater number of clinical variables known to be significant risk factors for COVID-19 outcomes. Consequently, our model may offer more advantages when employed in resource-limited areas.

While the WHO has lifted the pandemic status for COVID-19, this study aims to emphasize the importance of not forgetting the lessons learned from the past. Specifically, the dire need for utilizing artificial intelligence to enhance triaging capabilities, particularly during the peak of the pandemic when resources and manpower were scarce, should be highlighted.

## Limitations

This study has several limitations. Firstly, the number of subjects included in both the derivation and validation cohorts is relatively small. Secondly, the study utilized retrospective data, highlighting the need for prospective validation. Thidrly, we did not utilize control group. Finally, we did not analyse the impact of COVID-19 variants on the discriminative ability of the IMPACT scoring system.

## Conclusion

Our multicentre study introduces the Integrated Inpatient Mortality Prediction Score for COVID-19 (IMPACT), a clinical risk tool that integrates clinical, laboratory, and CXR data. This study provides valuable evidence on the real-world application of AI in clinical settings, demonstrating its potential for enhancing decision-making and improving patient care. The derivation and validation of IMPACT highlight the transformative role of AI in healthcare, enabling more personalized and effective treatment strategies for COVID-19 patients. Our research aims to bridge the gap between AI advancements and practical use, facilitating wider adoption of AI-based tools and revolutionizing disease prognostication in healthcare. However, it is imperative to conduct prospective validation of our findings, preferably utilizing a control group and extending the application to broader populations.

### Supplementary Information

Supplementary Information.

## Data Availability

Data is available upon a reasonable request to corresponding author.

## Abbreviations

AI: Artificial intelligence
ALA: Affected lung area
AUROC: Area under the receiver operating curve
CXR: Chest X-ray
CT-SS: Computed tomography severity score
IMPACT: Integrated inpatient mortality prediction score for COVID-19
LASSO: Least absolute shrinkage and selection operator
LOC: Loss of consciousness
MAP: Mean arterial pressure
MICE: Multiple imputation by chained equations
NETs: Neutrophil extracellular traps
PACS: Picture archiving and communication system
RT-PCR: Real-time reverse-transcription polymerase-chain-reaction
SPSS: Statistical program for social science
SpO2: Peripheral oxygen saturation

## Code availability

Please note that the code used for CAD4COVID is proprietary and not publicly available. For inquiries or questions regarding CAD4COVID, kindly refer to the Delft Imaging website at https://www.delft.care/cad4covid/.
