# Supplementary material for: Derivation and validation of novel integrated inpatient mortality prediction score for COVID-19 (IMPACT) using clinical, laboratory, and AI—processed radiological parameter upon admission: a multicentre study
Source: Sci Rep. 2024 Jan 25;14:2149. doi: 10.1038/s41598-023-50564-9 (PMC10810804; doi:10.1038/s41598-023-50564-9)

Table S1. Area under the receiver operating curve (AUROC) comparisons of IMPACT scoring system stratified according to the year of admission. The comparisons were performed using the DeLong’s test.

| **AUROC comparisons** | **P Value** |
| --- | --- |
| Total vs 2020 | 0.2862 |
| Total vs 2021 | 0.5685 |
| Total vs 2022 | 0.8123 |
| 2020 vs 2021 | 0.1731 |
| 2020 vs 2022 | 0.7523 |
| 2021 vs 2022 | 0.6793 |

**STEP-BY-STEP MODEL CONSTRUCTION**

**Step 1**


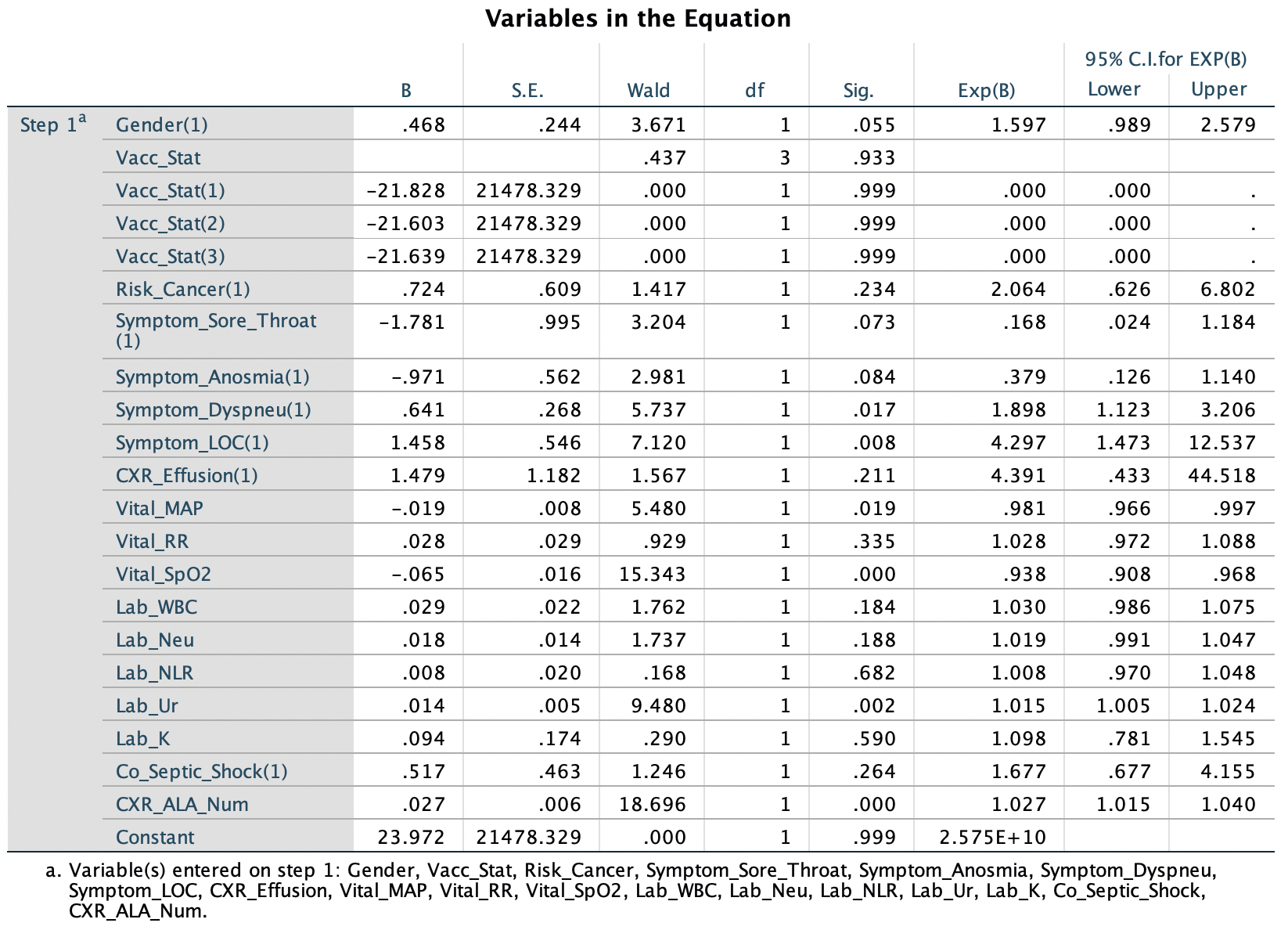


**Step 2**

**
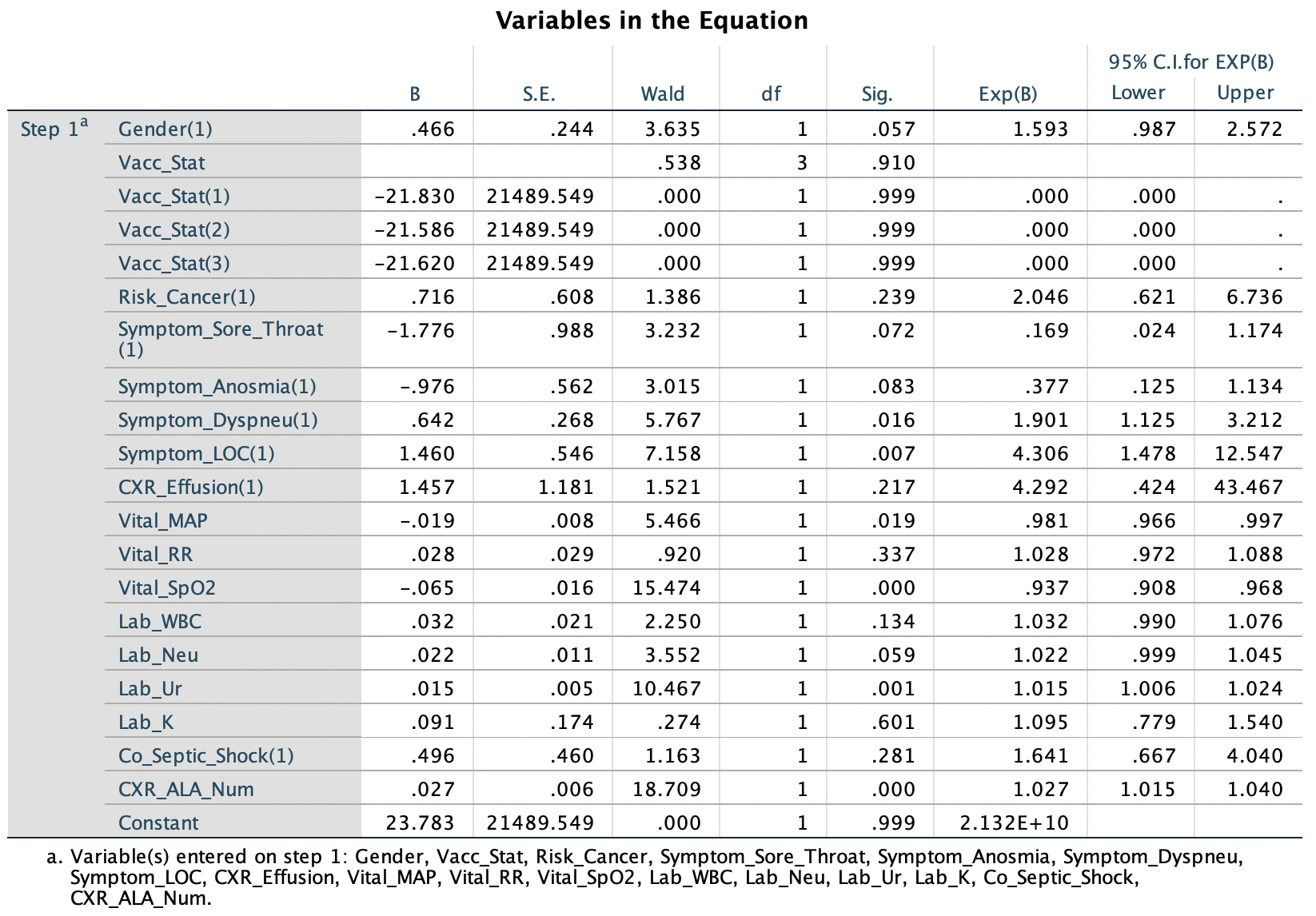
**

**Step 3**


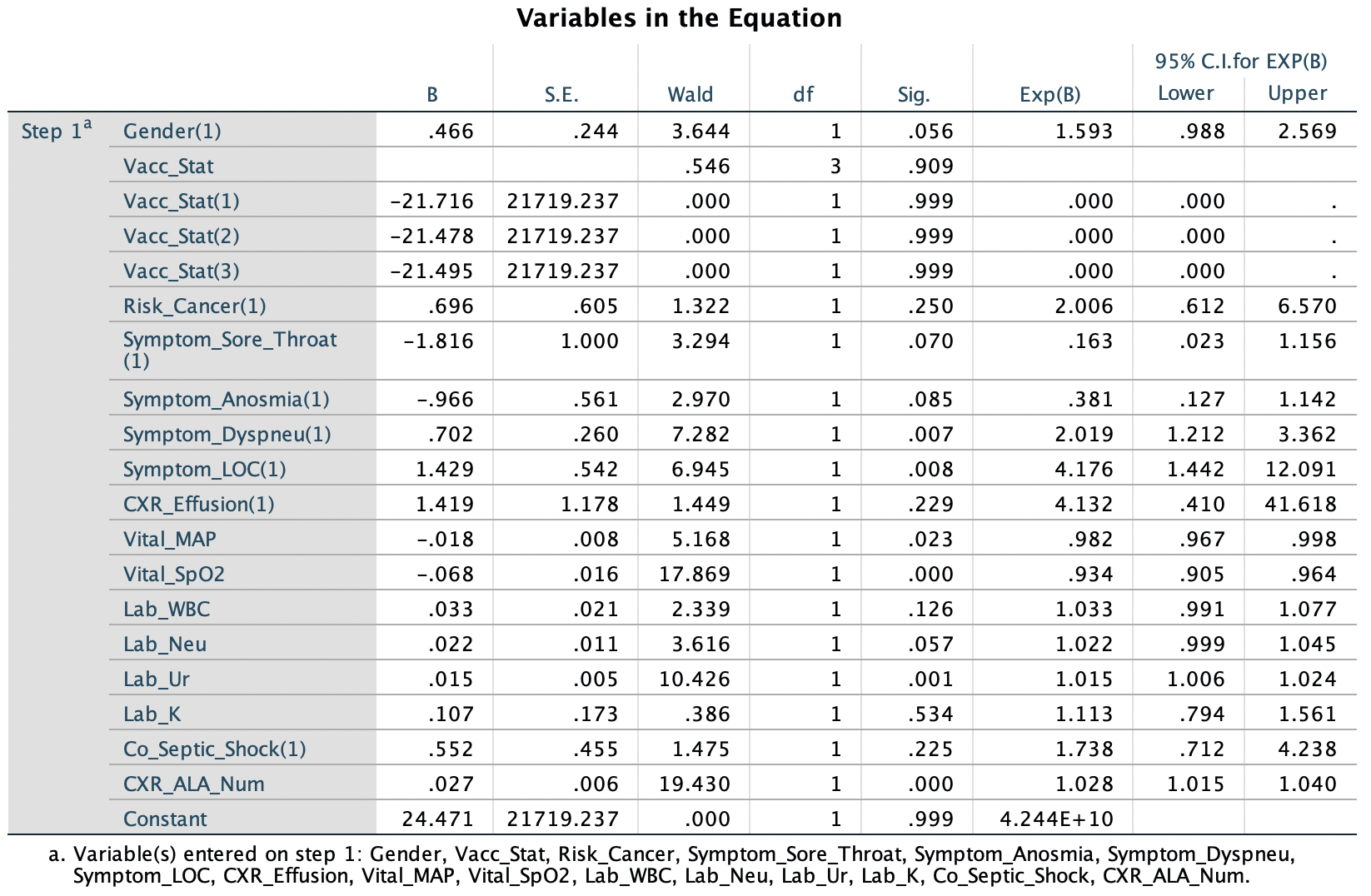


**Step 4**

**
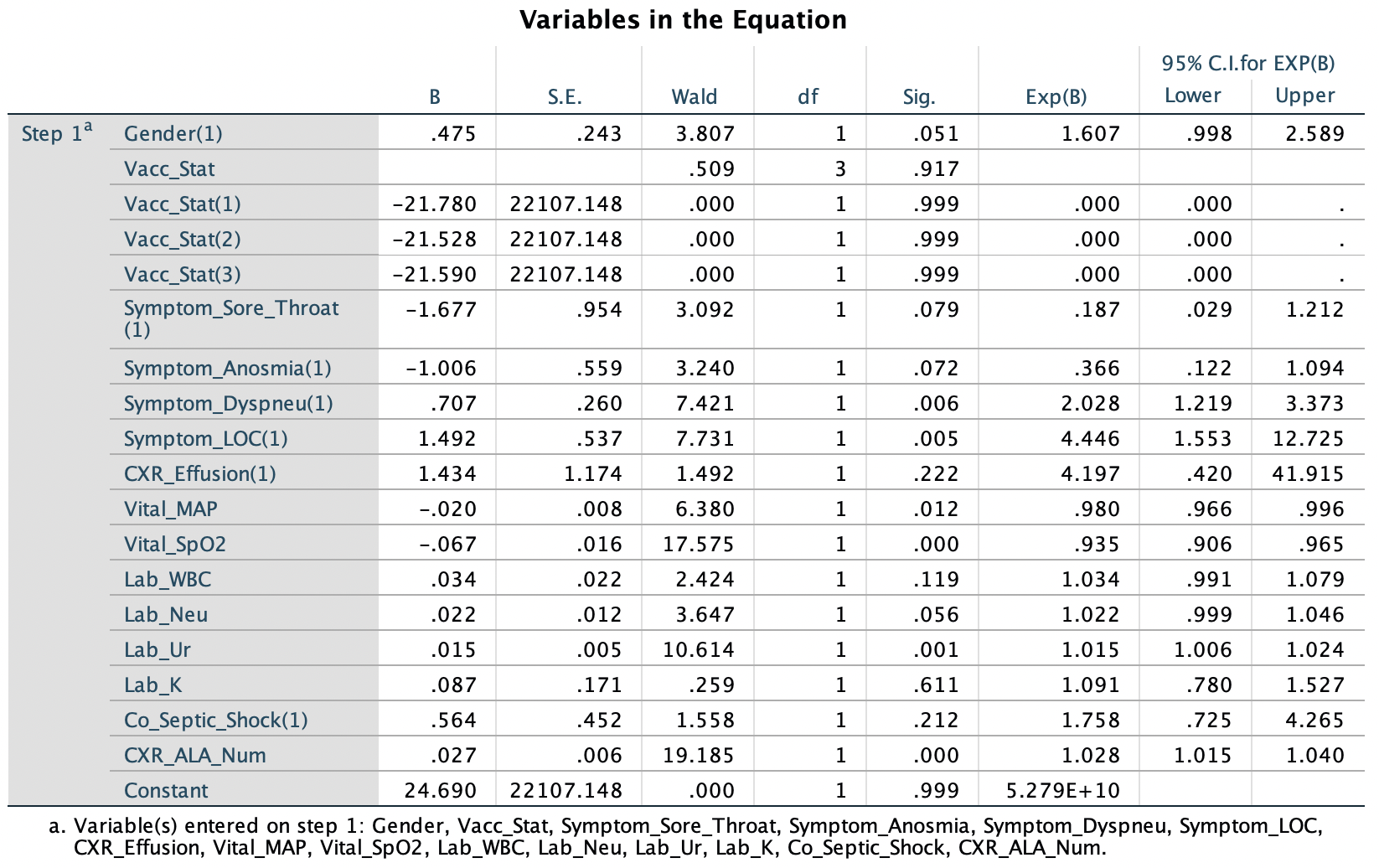
**

**Step 5**


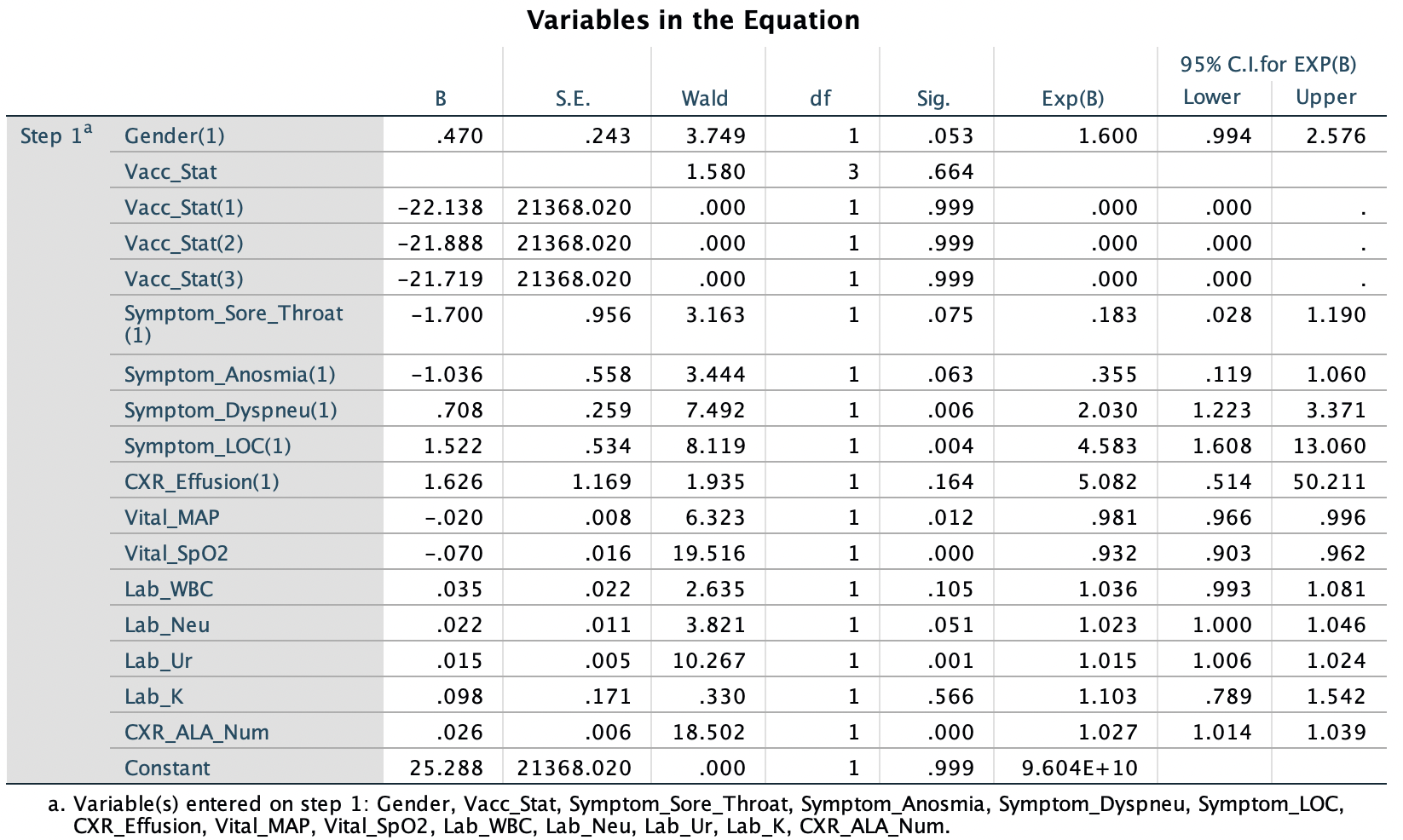


**Step 6**


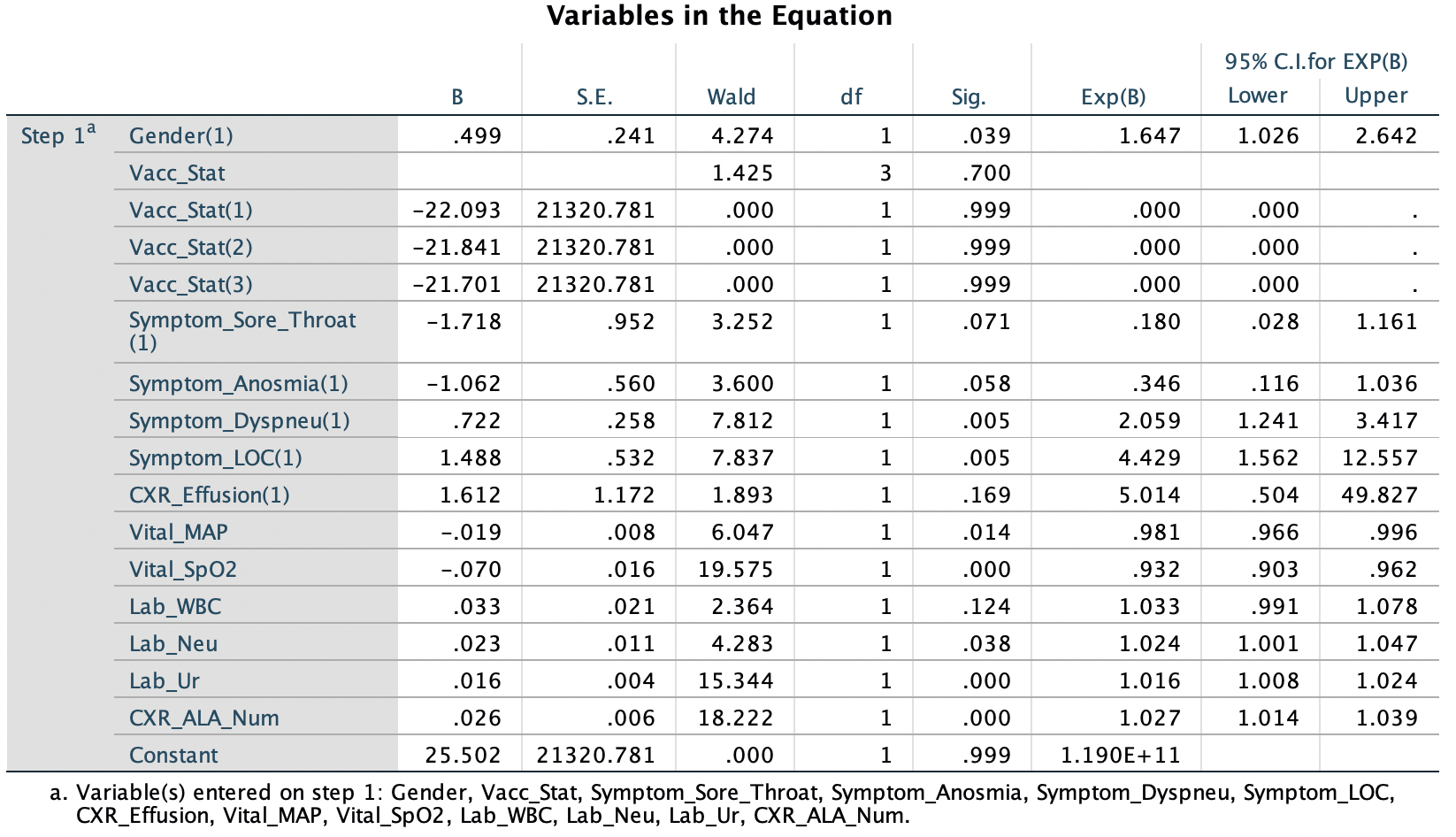


**Step 7**

**
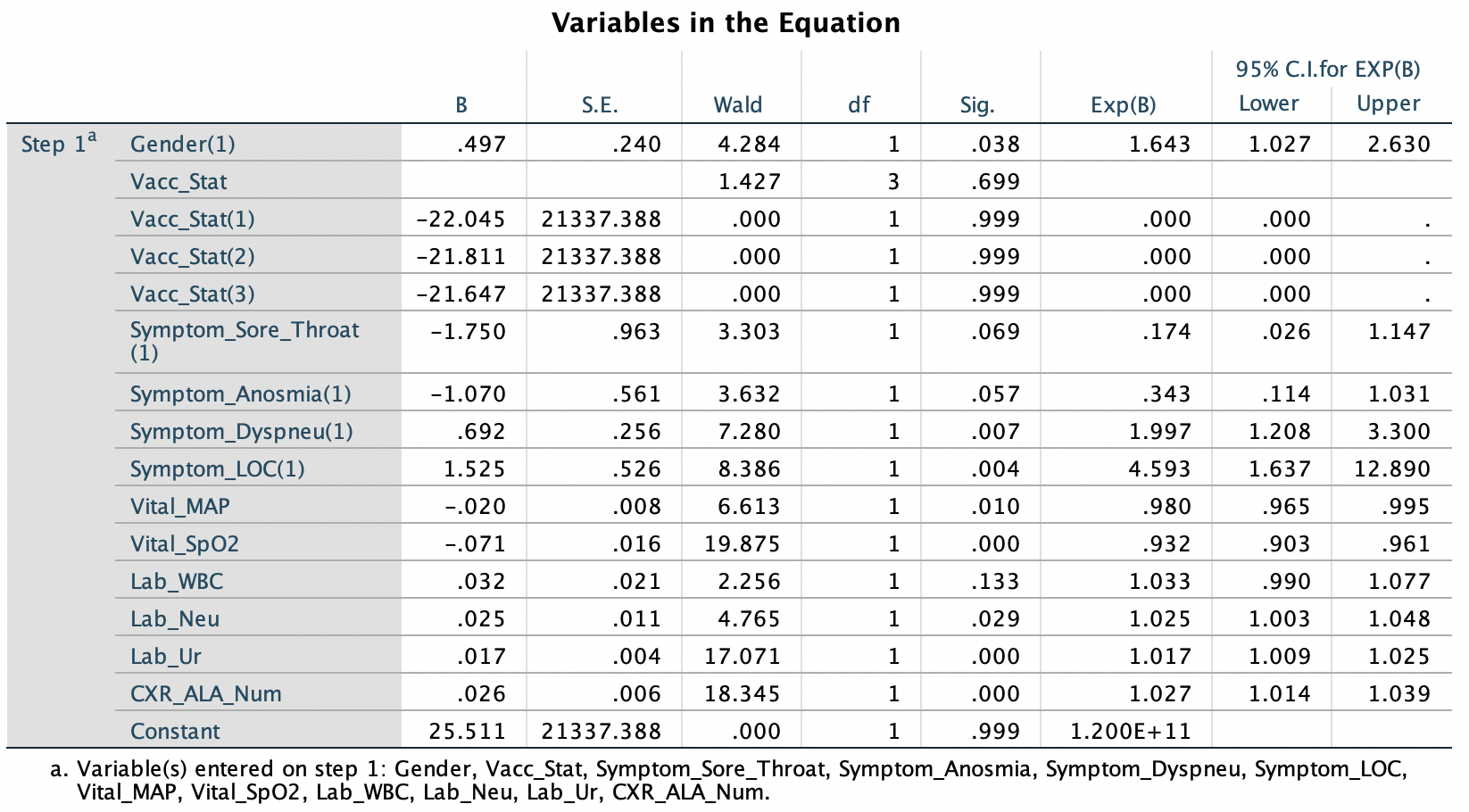
**

**Step 8**
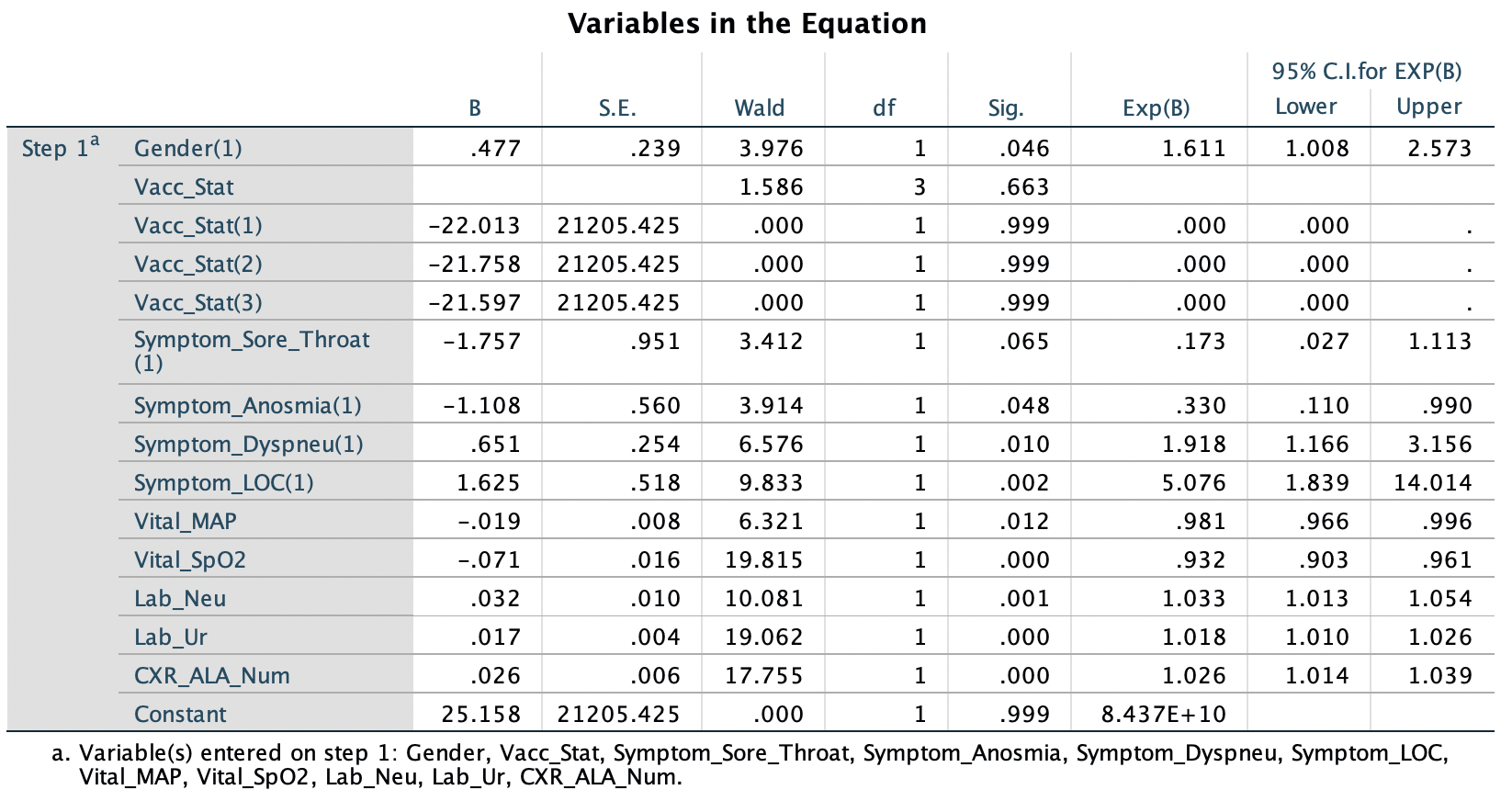


**
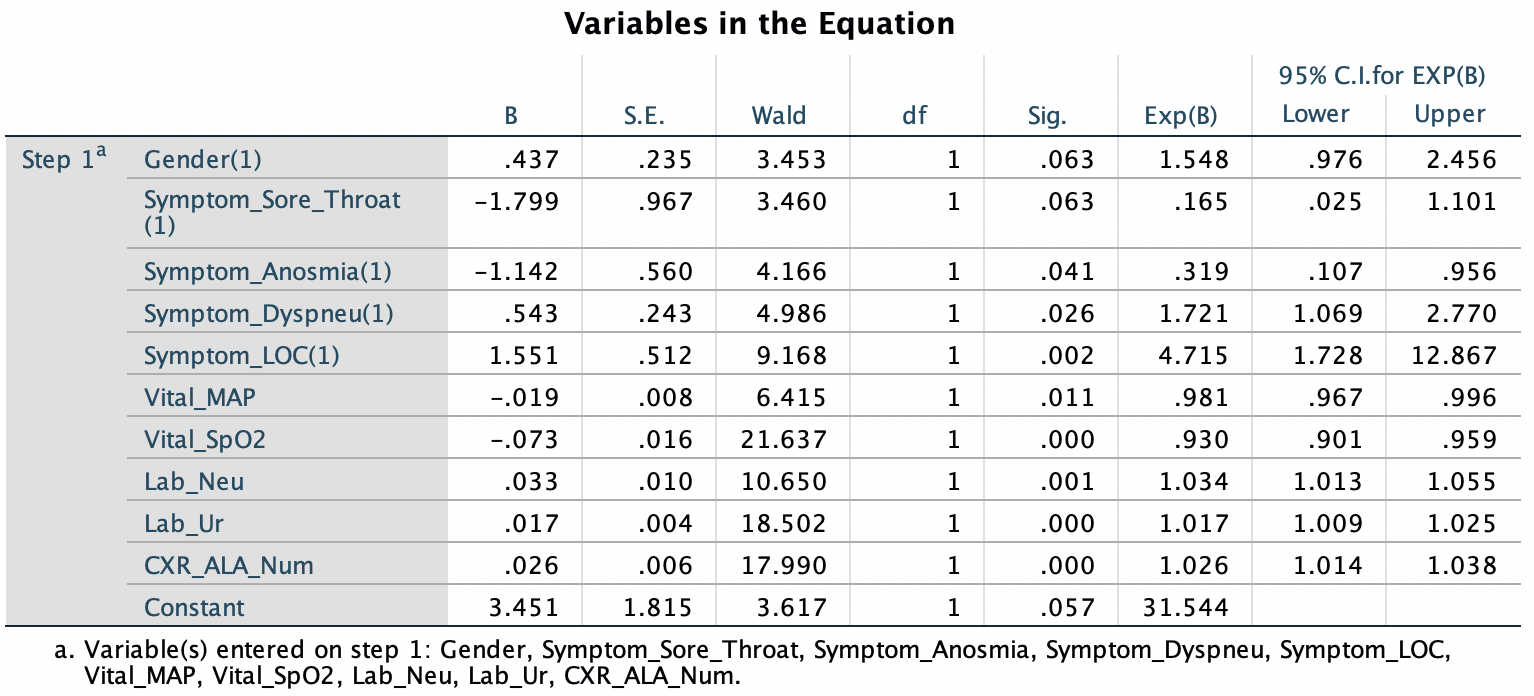
Step 9**

**Step 10**


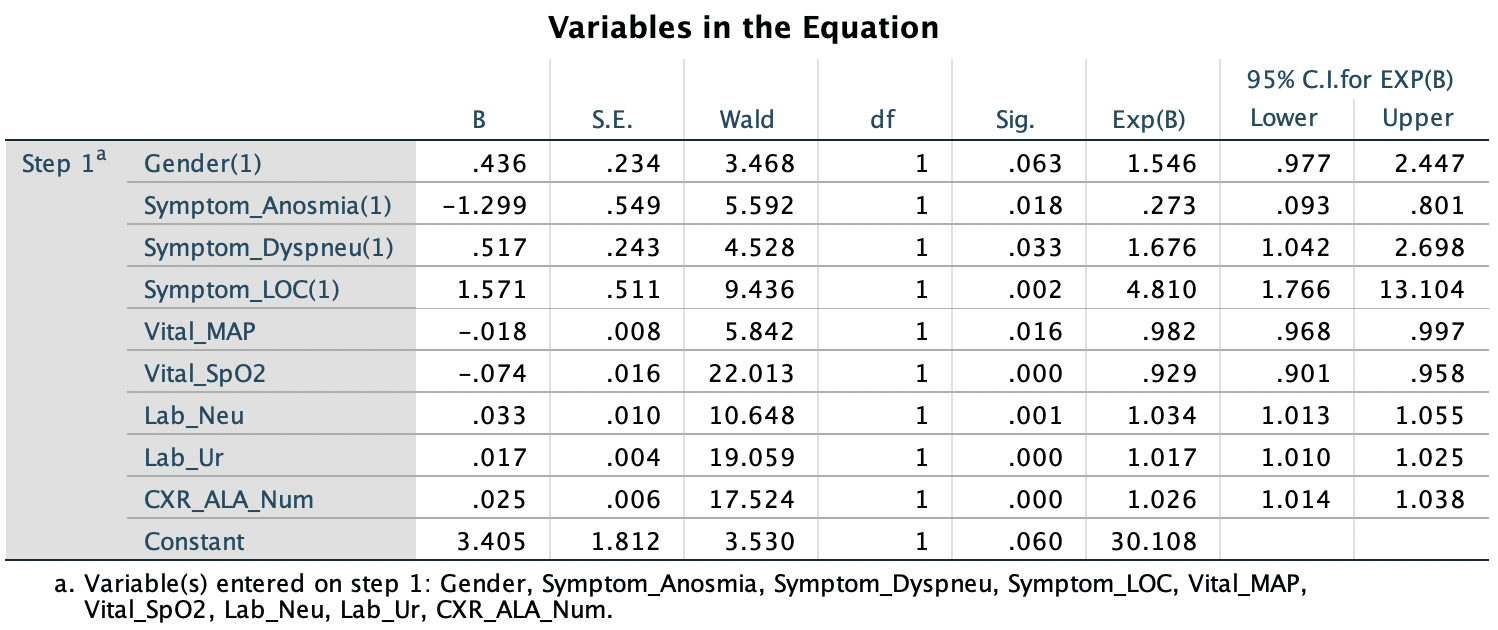

Supplement: Supplementary file 1 — Supplementary Information. [file 41598_2023_50564_MOESM1_ESM.docx]
